# Complete Caudate Lobectomy: Its Definition, Indications, and Surgical Approaches

**DOI:** 10.1155/1998/92312

**Published:** 1998-12

**Authors:** Akinori Sasada, Keiji Ataka, Kazuhiko Tsuchiya, Hiroyuki Yamagishi, Hiromi Maeda, Masayoshi Okada

**Affiliations:** 1 Department of Surgery Division II Kobe University School of Medicine Kobe Japan; 2 Department of Surgery Division II Kobe University School of Medicine 7-5-2, Kusunoki-cho Chuo-Ku Kobe 650 Japan

## Abstract

There are three ways to approach and resect the caudate lobe of the liver, that is; and isolated caudate lobectomy, a combined resection of the liver overlying the caudate lobe, and a transhepatic anterior approach by splitting parenchyma of the liver.

We had two patients with neoplasms originating in the caudate lobe who underwent a complete caudate lobectomy. Both patients have been doing well without liver dysfunction. Although after the transhepatic anterior approach we anticipated an adverse effect from splitting the parenchyma of the liver, the postoperative course was uneventful and similar to that of the right side approach.


*HPB Surgery*, 1998, Vol. 11, pp. 87–95
Reprints available directly from the publisher
Photocopying permitted by license only

© 1998 OPA (Overseas Publishers Association) N.V.
Published by license under
the Harwood Academic Publishers imprint,
part of The Gordon and Breach Publishing Group.
Printed in Malaysia.

### Complete Caudate Lobectomy: Its Definition, Indications, and Surgical Approaches

AKINORI SASADA*, KEIJI ATAKA , KAZUHIKO TSUCHIYA, HIROYUKI YAMAGISHI, HIROMI MAEDA and MASAYOSHI OKADA

*Department of Surgery Division II, Kobe University School of Medicine, Kobe, Japan*

*(Received 12 March 1997; In final form 30 September 1997)*

**There are three ways to approach and resect the caudate lobe of the liver, that is; and isolated caudate lobectomy, a combined resection of the liver overlying the caudate lobe, and a transhepatic anterior approach by splitting parenchyma of the liver.**

**We had two patients with neoplasms originating in the caudate lobe who underwent a complete caudate lobectomy. Both patients have been doing well without liver dysfunction. Although after the transhepatic anterior approach we anticipated an adverse effect from splitting the parenchyma of the liver, the postoperative course was uneventful and similar to that of the right side approach.**

*Keywords:* Complete caudate lobectomy, neoplasm originating, in caudate lobe, transhepatic anterior approach, right side approach

### INTRODUCTION

Although it has been demonstrated that various hepatic resections was carried out safely [1, 2], the caudate lobectomy is still a challenging problem because of its anatomical complexity [3].

The caudate lobe is easily invaded by cancer of the hilar bile ducts, so that the necessity of combined resection of the caudate lobe has been emphasized in curative operation for cancer of the hilar bile ducts [4, 5]. While the caudate lobectomy for neoplasm originating in the caudate lobe itself is very rare, and this procedure frequently causes a state of confusion because of the anatomical difficulty.

In this report we have defined the complete caudate lobectomy as the whole resection of the spigelian lobe, the paracaval portion and the caudate process [3], and described here how to approach the caudate lobe and our surgical experiences of the complete caudate lobectomy.

### Surgical Anatomy

The caudate lobe consists of the spigelian lobe (the left lobe, or segment I in Couinaud's classification), the paracaval portion (the right lobe, or segment IX in Couinaud's classification [6]) and the caudate process (Fig. 1). The caudate lobe has some variations of its vascular structure [3, 7]. The arterial branches arise from the left

*Address for reprint requests: Akinori Sasada, MD, Department of Surgery Division II, Kobe University School of Medicine, 7-5-2, Kusunoki-cho, Chuo-Ku, Kobe, 650, Japan.

caval portion of the caudate lobe with or without imparied liver function.

### Patients and Methods

During the last last five years, we have operated on two patients with neoplasms originating in the caudate lobe, one large hemangioma and one metastatic liver carcinoma from a rectal carcinoma.

The preoperative liver function was within normal limits in each patient, and the hepatic parenchyma in each patient was also macroscopically normal without chromic degenerative changes.

The complete caudate lobectomy for these two patients is summarized in Table I.

*Transphepatic anterior approach* was chosen in a 36 year-old female patient complaining of continuous back pain with a large hemangioma in the caudate lobe (Fig. 2a) because the parent was young and their would be minimal loss of the remaining liver.

The laparotomy was performed through a J-shaped abdominothoracic incision with a diaphragmatic transection.

On inspection, the left portion of the spigelian lobe remained normal. The intraoperative ultra-

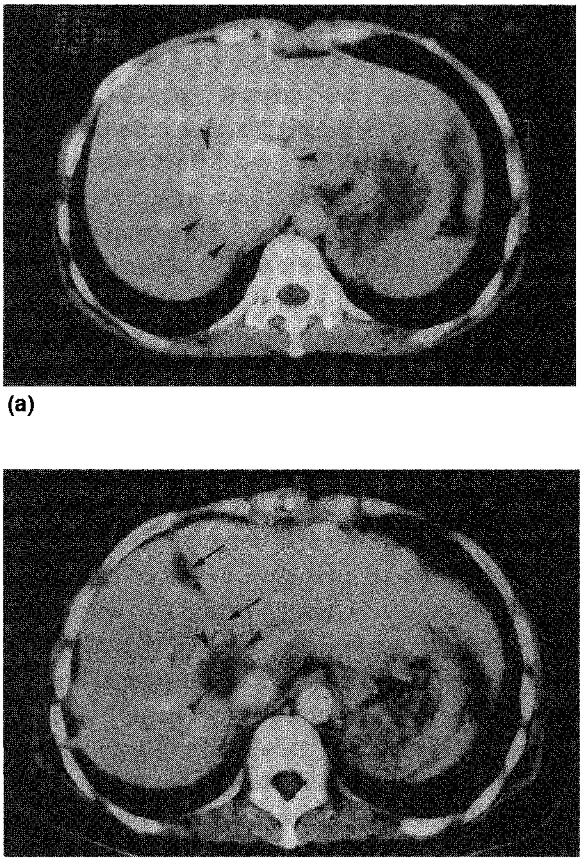

(a) (b)

FIGURE 2

TABLE I Complete caudate lobectomy for neoplasm originating in the caudate lobe

| Patient No | 1 | 2 |
|---|---|---|
| Age/sex | 36/F | 50/M |
| gross pathology | hemangioma | metastatic liver tumor |
| approach to caudate lobe | anterior | right |
| combined resection | none | posterior segment |
| occulsion time of hepatic inflow | 90 min | 55 min |
| vascular occlusion | Pringle's maneuver | hemihepatic vascular occlusion |
| Operation time | 11 hrs 40 min | 7 hrs 30 min |
| location of tumor in caudate lobe |  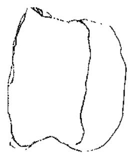 |  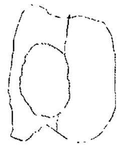 |
| size of tumor | 7.0 × 5.0 × 3.0 cm | 3.0 × 1.8 × 2.0 cm |
| blood loss | 5,600 ml | 1,600 ml |
| blood transfusion | 3,000 ml | none |
| outcome | 3 y, alive | 1 y, no recurrence alive |

sonography demonstrated that the hemangioma entirely occupied the paracaval portion and caudate process. The cranial extremity of hemangioma was situated at the confluence of right hepatic vein (RHV) and MHV that drained into the IVC. The caudal extremity protruded below the hepatic hilum. The hemangioma displaced the IVC posteriosly. After complete mobilization of both hepatic lobes, the ligamentum venosum between the left hepatic vein and left portal vein was resected. The liver was then isolated from both sides of the IVC by dividing and dissecting SHVs. By the hepatic hilar preparation, glissonian vessels into the portal vein (PV) were divided. The bile duct and the hepatic artery were each encircled with a tape, and then the hilar branches to the caudate lobe were dissected. Under Pringle's maneuver (clamping for 15 minutes and declamping for 5 minutes), hepatic transection started along left wall of the MHV toward the hepatic hilum. Thereafter the hepatic parenchyma was split in two directions; on the left side toward the resected ligamentum venosum and on the right side just behind the MHV.

In order to identify the boundary between the right hepatic lobe and the right border of the paracaval portion and caudate process, 15 ml of indigotindisulfonate sodium solution was injected into the right posterior branch of the PV. The right border of the caudate lobe was thereby kept unstained and was marked along the unstained margin with an electrocautery. The complete caudate lobectomy was performed *en bloc* from caudally to cranially. The bleeding from the liver raw surface continued until complete removal of the hemangioma occupying the paracaval portion, because its cranial extremity was situated at the confluence of the RHV and MHV into the IVC. The operation time was 11 hours 40 minutes, and intraoperative blood transfusion was required for the blood loss of 5,600 ml. The removed hemangioma was $7.0 \times 5.0 \times 3.0$ cm in size and histology revealed cavernous hemangioma.

*Right side approach* was used in a 50 year-old male patient who underwent Miles' operation for rectal carcinoma three years previously, and was recently found to have metastatic lesions in the caudate lobe and segment S6 (Fig. 3a).

A thoracoabdominal incision through the right seventh intercostal space was made. The diaphragm was diagonally transected towards the IVC.

Intraoperative ultrasonography of the whole liver revealed no other metastatic lesion. Therefore, we decided to carry out the complete caudate lobectomy concomitant with the right posterior segmentectomy by means of a right side approach.

After complete mobilization of both hepatic lobes from the abdominal wall and the diaphragm, the gallbladder was removed to expose the hepatic hilum. The mobilized lateral segment was lifted ventrally to separate the ligamentum venosum. Since the fissure for the ligamentum venosum was completely separated, the left border of the spigelian lobe was freed from the IVC. The common hepatic duct (CHD) was separated and encircled with a tape. By retracting the tape around the CHD to the left and upward, the right hepatic artery behind the CHD was encircled with a tape. The surrounding connective tissue along the right hepatic artery was dissected until its division into the anterior and posterior branches. Subsequently the right portal vein (RPV) was separated to be encircled with a tape, and was dissected along the RPV up to its bifurcation into the anterior and posterior branches.

Each branch of the RPV was encircled with a tape, respectively. The vessels to the left lobe of the liver were also separated to be encircled with a tape as in the right lobe. The hilar separation of arterial and portal branches to the caudate lobe was performed. The SHVs were completely ligated and dissected from both sides of the IVC.

Next the right posterior branches of the hepatic artery and portal vein were ligated, the demarcated area appeared on the surface of the liver.

We confirmed the RHV by ultrasonography, a transection line on the liver, at the discolored border, was marked on the surface of the liver with an electrocautery.

A hemihepatic vascular occlusion (clamping for 30 minutes and declamping for 10 minutes) was applied to control the blood supply to the right remnant lobe of the liver. The transection along the intersegmental plane of the right lobe was continued toward the freed ligamentum venosum. Half way through the transection, each posterior segmental branch of the hepatic artery, portal vein and bile duct was ligated doubly and dissected at the hepatic hilum.

As the posterior segment of the liver was retracted lateral-ventrally, the complete caudate lobectomy together with a right posterior segmentectomy of the liver was performed as en bloc.

The operation time was 7 hours 30 minutes, but no blood transfusion was needed. The resected liver weighed 190 gm and contained two metastatic tumors. One lesion of $3.0 \times 1.8 \times 2.0$ cm mainly occupied the paracaval portion and caudate process, and the other of $3.0 \times 1.5 \times 1.5$ cm in size located in S6. The histological reports of both lesions were adenocarcinoma derived from the previous rectal carcinoma.

### POSTOPERATIVE COURSE

The postoperative courses were uneventful in both patients without any signs of liver failure. The postoperative peak levels of total bilirubin were 35.7 µmol/l and 34 µmol/l on the first postoperative day, respectively, and then gradually returned to normal values. The anticipated adverse effect from splitting the liver parenchyma was hardly recognized in Patient 1.

Postoperative computed tomography after one month in Patient 1 demonstrated the cavity around the IVC and the free space along the split line of the liver parenchyma (Fig. 2b). The follow-up magnetic resonance imaging (MRI) after 6 months of the operation in Patient 2 showed the small cavity around the IVC substituting the resected caudate lobe. The remnant liver was well hypertrophied. No recurrent lesions were detected on the MRI (Fig. 3b).

Both patients have been doing well without liver dysfunction nor recurrence after 3 years and 1 year respectively, after complete caudate lobectomy.

### DISCUSSION

A neoplasm originating in the caudate lobe is very rate [16], so that an isolated complete

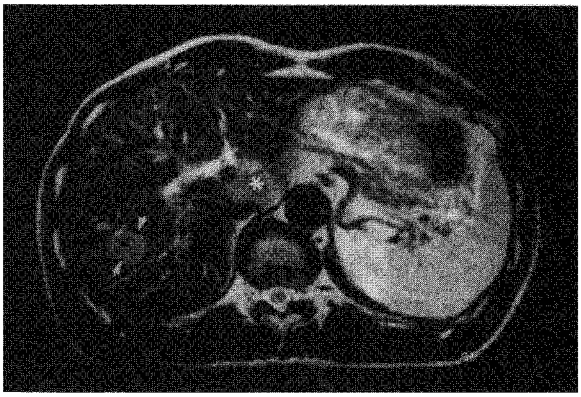

(a)

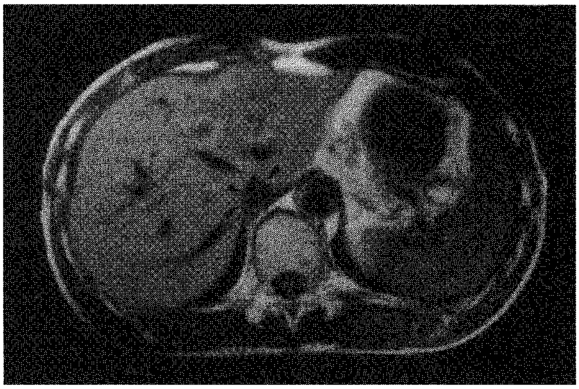

(b)

FIGURE 3

resection of the caudate lobe still remains unfamiliar to many surgeons.

Our cases were one hemangioma, and one metastatic tumor from rectal carcinoma and the complete caudate lobectomy was successfully performed through the transhepatic anterior approach and the right side approach combined with a posterior segmentectomy, respectively.

When a tumor is too large and mainly located in the paracaval portion of the liver, the transhepatic anterior approach is generally used to perform the precise surgery [15], although this surgical procedure is more complex than other approaches.

The right border of the paracaval portion, which lies in front and to the right of the IVC, is in continuity with the right lobe of the liver. Furthermore, between the IVC and the right portal pedicle, the caudate lobe is united with the right posterior segment of the liver by the caudate process. As it is hard to recognize the landmark indicating the boundary of the caudate lobe and the right lobe of liver, we used to counterstaining method [17] with injection of the dye into the posterior branch of the PV. The paracaval portion receives a blood supply from many tiny vessels other than the posterior segmental branch [6]. Therefore, a counterstaining the posterior segment of the liver is impractical but useful clinically [15, 17].

In comparison with conventional procedures, the transphepatic anterior approach has some disadvantages of longer operative duration, more blood loss and harmful effects by splitting parenchyma of the liver. We thought that the resection through the caudal approach would be more dangerous in this case, because this approach gives a very narrow surgical field, and moreover, the hemangioma in Patient 1 occupied mainly the paracaval portion and caudate process, its cranial extremity reached the confluence of the RHV and MHV into the IVC. Indeed, and intraoperative hemorrhage continued until complete removal of the tumor, requiring intraoperatively 3,000 ml of blood transfusion for 5,600 ml of blood loss. However, the postoperative liver function was smoothly restored to normal range in a few days without harmful effects from splitting the liver parenchyma in half.

In spite of the demerits described above, the transhepatic anterior approach, providing an excellent surgical view, is safe and gives good access to perform a complete resection of the caudate lobe [14, 15]. This approach also preserves the remnant liver function well as in our patient.

The right side approach for the caudate lobectomy is carried out to simplify the procedure [8, 13] or to omit dissection of the unclear right caudal border. The hepatic transection through the intersegmental plane along dorsal aspect of the RHV using hemihepatic vascular occlusion was simpler anatomically and technically than a transhepatic anterior approach. Therefore, this procedure had a shorter operating time and less blood loss. However, a preparatory or combined resection of the hepatic segment or lobe overlying the caudate lobe could trigger off postoperative hepatic failure even in the normal liver [1, 15]. All the more for the patient with impaired liver function, a combined resection and sacrifice of hepatic parenchyma could easily cause fatal hepatic failure.

Recently some authors [10–12] described novel procedures about an isolated caudate lobectomy. However, their procedures would be still critical and risky because of the anatomical complexity and a poor opportunity for surgical manipulation in the limited space behind the main liver lobes. Therefore, an *ex situ* operation has been occasionally recommended instead of these difficult procedures [8, 18].

We performed the transhepatic anterior approach and the right side approach for the complete caudate lobectomy in two patients. Both approaches had excellent results without any troubles. Finally, we would emphasize that surgeons must give a serious consideration on a patient by patient basis including the occupied region of tumor, the remnant liver reserve and the vascular structure surrounding tumor in order to determine the best approach to the caudate lobe.

### References

[1] Starzl, T. E., Waterman, P. M., Thiel, V. D., Diliz, P. H. S., Dekker, A. and Bron, K. M. (1982). Left hepatic trisegmentectomy. *Surg. Gynecol Obstet.*, **115**, 21-27.

[2] Makuuchi, M., Hashikura, Y., Kawasaki, S., Tan, D., Kosuge, T. and Takayama, T. (1993). Personal experience of right anterior segmentectomy (segment V and VIII) for hepatic malignancies. *Surg.*, **114**, 52-58.

[3] Kumon, M. (1985). Anatomy of the caudate lobe with special reference of portal vein and bile duct (in Japanese with English abstract). *Acta. Hepatol. Jpn.*, **12**, 1193-1199.

[4] Mizumoto, R., Kawarada, Y. and Suzuki, H. (1986). Surgical treatment of hilar carcinoma of the bile duct. *Surg. Gyncol. Obstet.*, **162**, 153-158.

[5] Nimura, Y., Hayakawa, N., Kamiya, J., Kondo, S. and Shionoya, S. (1990). Hepatic segmentectomy with caudate lobe resection for bile duct carcinoma of the hepatic bilus. *World J. Surg.*, **14**, 535-544.

[6] Couinaud, C. (1994). The paracaval segments of the liver. *J. Hep. Bil. Pancr. Surg.*, **2**, 145-151.

[7] Nimura, Y., Hayakawa, N., Kamiya, J., Kondo, S., Nagino, M. and Kanai, M. (1995). Hilar Cholangiocarcinoma-surgical anatomy and curative resection. *J. Hep. Bil. Pancr.*, **2**, 239-248.

[8] Elias, D., Lasser, P. H., Desruennes, E., Mankarios, H. and Jiang, Y. (1992). Surgical approach to segment I for malignant tumors of the liver. *Surg. Gynecol. Obstet.*, **175**, 17-24.

[9] Lerut, J., Gruwez, J. A. and Blumgart, L. H. (1990). Resection of the caudate lobe of the liver. *Surg. Gynecol. Obstet.*, **171**, 160-162.

[10] Colonna, J. O., Shaked, A., Gelabert, H. A. and Busuttil (1993). Resection of the caudate lobe through "bloody gulch", *Surg. Gynecol. Obstet.*, **176**, 401-402.

[11] Yanaga, K., Matsumata, T., Hayashi, H., Shimada, M., Urata, K. and Sugimachi, K. (1994). Isolated hepatic lobectomy, *Surg.*, **115**, 757-761.

[12] Takayama, T., Tanaka, T., Higashi, T., Katou, K., Teshima, Y. and Makuuchi, M. (1994). High dorsal resection of the liver. *J. Am. Coll. Surg.*, **179**, 73-75.

[13] Miller, C. M., Schwartz, M. E. and Nishizaki, T. (1991). Combined hepatic and vena caval resection with autogenous caval graft replacement. *Arch. Surg.*, **126**, 106-108.

[14] Yamamoto, J., Takayama, T., Kosuge, T., Yoshida, J., Shimada, K., Yamasaki, S. and Hasegawa, H. (1992). An isolated caudate lobectomy by the transhepatic approach for hepatocellular carcinoma in cirrhotic liver. *Surg.*, **111**, 699-702.

[15] Kosuge, T.,Yamamoto, J., Takayama, T., Shimada, K., Yamasaki, S., Makuuchi, M. and Hasegawa, H. (1994). An isolated, complete resection of the caudate lobe, including the paracaval portion, for hepatocellular carcinoma. *Arch. Surg.*, **129**, 280-284.

[16] Tung, T. T. (1979). Bilan d'une experience: les resections majeures et mineures du foie. In: Tung, T. T. (Ed) Paris. Masson., p. 127.

[17] Takayama , T., Makuuchi, M., Watanabe, K., Kosuge, T., Takayasu, K., Yamazaki, S. and Hasegawa, H. (1990). A new method for mapping hepatic subsegment: Counterstaining identification technique. *Surg.*, **109**, 226-229.

[18] Pichlmayr, R., Grosse, H., Hauss, J., Gubernatis, G., Lamesch, P. and Bretschneider, H. J. (1990). Technique and preliminary results of extracorporeal liver surgery (bench procedure) and of surgery on the *in situ* perfused liver. *Br. J. Surg.*, **77**, 21-26.

### INVITED COMMENTARY

### J. Belghiti

Dr Sasada and his associates are to be congratulated for publishing two complete caudate lobectomies using respectively a transhepatic anterior approach and a right side approach. This succcessful result is the result of an excellent knowledge of the segmental anatomy of the liver an an impressive mastering of liver resection. This paper contains one of the best illustration of the anatomy of the caudate lobe ever published. A good knowledge of this hidden part of the liver which is of extreme importance in liver surgery because it can be involved by tumours. The so-called dorsal sector by Couinaud consists of segment I, or the caudate lobe, and segment IX, also named the right paracaval region [1]. This dorsal sector is situated between the IVC and the large (right, middle and left) hepatic veins and the liver hilum. Japanese authors refer to this entire part simply as the caudate lobe and divide it into a left part, or Spiegel's lobe, and a right part [2]. A surgeon should know that he can see and palpate only the left part of this dorsal sector, but a large part of this region of the liver is hidden. Dr Sasadas' description of the access of the right part of the dorsal sector through a transhepatic anterior approach in one case and after complete mobilisation of the right liver should be noticed. According to our experience, further studies should determine the indication of primary resection of the left lateral segment (segment I and II) in order to facilitate the access to the dorsal sector. As experienced by the authors the situation of the dorsal sector between the IVC and the large hepatic veins may cause major bleeding. In this indication we advocate total vascular occlusion of the liver [3]. Although we dramatically restrict our indications of *ex-situ* procedure, we think that this method should be considered in such cases [4].

### *References*

[1] Couinaud, C. (1994). The paracaval segments of the liver *J. Hepat Bil Pancer Surg.*, **2**, 145-151.

[2] Kosuge, T., Yamamoto, J., Takayama, T., Shimada, K., Yamasaki, S., Makuuchi, M. and Hasegawa, H. (1994). An isolated, complete resection of the caudate lobe, including the paracaval portion, for heparocellular carcinoma. *Arch. Surg.*, **129**, 280-284.

[3] Belghiti, J., Noun, R., Zante, E., Ballet, Th, Sauvanet, A. (1996). Portal triad clamping or hepatic vascular exclusion for major liver resection: controlled study. *Ann. Surg.*, **224**, 155-161.

[4] Sauvanet, A., Dousset, B. and Belghiti (1994). A simplified technique of "*ex-situ*" liver surgery. *The Journal of the American College of Surgeon*, **178**, 79-82.

J. Belghiti
Department of Digestive
Surgery and Transplantation
Hôpital Beaujon
University Paris VII

### COMMENTARY ON MANUSCRIPT – COMPLETE CAUDATE LOBECTOMY: ITS DEFINITION, INDICATIONS, AND SURGICAL APPROACHES

This paper describes two different approaches for complete caudate lobectomy and the experience gained when using the anterior, transhepatic and the right-sided approaches. Complete caudate lobectomy is uncommon and can be technically demanding. The present paper adds to existing experience [1-7].

The caudate lobe may be excised with a posterior (caudal, dorsal) approach or an anterior, transhepatic approach. It appears that a posterior approach is advisable in most patients, and that a transhepatic approach may be reserved for large tumours and especially for cirrhotic livers. Bleeding from hepatic veins is the main concern during caudate lobectomy, and this may be difficult to avoid or control unless the liver, including the caudate lobe, can be fully mobilized. It may be impossible to safely obtain full mobilization and control with the posterior approach if the tumour is large and firm and especially if the liver is rigid from cirrhosis. A transhepatic approach may therefore be recommended in these situations. In some patients with liver cirrhosis it is, however, possible to perform a safe posterior resection if the hepatic veins can be controlled extrahepatically [5-7]. Extrahepatic control of the hepatic veins is advisable also in patients without cirrhosis if the tumour is large and/or is close to the entrance of the (right), middle and left hepatic veins into the vena cava and may be combined with the transhepatic approach. I have no experience with the transhepatic approach, but I guess that I would have enucleated the haemangioma (patient no. 1) using a posterior approach and control of hepatic veins.

Surgical strategy should of course be tailored to the circumstances. Among other things, this also means that the posterior approach can be righ-sided or left-sided or alternate between the two sides, as emphasized by Bartlett *et al.* [7]. The rule is to operate where it is as "easy" as possible.

The fact that isolated caudate lobectomy is technically feasible does not mean that it should be used in all patients with malignant tumour in the caudate lobe. As emphasized by Elias *et al.* [2], there is risk for inadequate tumor clearance for anatomical reasons. Thus, in a patient with adequate hepatic reserve one should not hesitate to remove more segments *en bloc* with the caudate lobe to ensure free resection margins.

### References

[1] Lerut, J., Gruwez, J. A. and Blumgart, L. H. (1990). Resection of the caudate lobe of the liver. *Surgery, Gynecology and Obstetrics*, **171**, 160–162.

[2] Elias, D., Lasser, P. H., Desruennes, E., Mankarios, H. and Jiang, Y. (1992). Surgical approach to segment I for malignant tumors of the liver. *Surgery, Gynecology and Obstetrics*, **175**, 17–24.

[3] Yamamoto, J., Takayama, T., Kosuge, T., Yoshida, J., Shimada, K., Yamasaki, S. and Hasegawa, H. (1992). An isolated caudate lobectomy by the transhepatic approach for hepatocellular carcinoma in the cirrhotic liver. *Surgery*, **111**, 699–702.

[4] Colonna II, J. O., Shaked, A., Gelabert, H. A. and Busuttil, R. W. (1993). Resection of the caudate lobe through "bloody gultch". *Surgery, Gynecology and Obstetrics*, **176**, 401–402.

[5] Takayama, T., Tanaka, T., Higaki, T., Katou, K., Teshima, Y. and Makuuchi, M. (1994). High dorsal resection of the liver. *Surgery, Gynecology and Obstetrics*, **179**, 72–75.

[6] Yanaga, K., Matsumata, T., Hayashi, H., Shimada, M., Urata, K., Sugimachi, K. (1994). *Surgery*, **115**, 757–761.

[7] Bartlett, D., Fong, Y. and Blumgart, L. H. (1996). Complete resection of the caudate lobe of the liver technique and results. *British Journal of Surgery*, **83**, 1076–1081.

Karl-G Tranberg
Department of Surgery
Lund Unversity
Lund, Sweden

